# Deep Learning of Speech Data for Early Detection of Alzheimer’s Disease in the Elderly

**DOI:** 10.3390/bioengineering10091093

**Published:** 2023-09-18

**Authors:** Kichan Ahn, Minwoo Cho, Suk Wha Kim, Kyu Eun Lee, Yoojin Song, Seok Yoo, So Yeon Jeon, Jeong Lan Kim, Dae Hyun Yoon, Hyoun-Joong Kong

**Affiliations:** 1 Interdisciplinary Program in Medical Informatics Major, Seoul National University College of Medicine, Seoul 03080, Republic of Korea; mikeahn@snu.ac.kr; 2 Department of Transdisciplinary Medicine, Seoul National University Hospital, Seoul 03080, Republic of Korea; chovis@snuh.org; 3 Medical Big Data Research Center, Seoul National University College of Medicine, Seoul 03080, Republic of Korea; kimsw@snu.ac.kr (S.W.K.); kyueunlee@snu.ac.kr (K.E.L.); 4 Department of Medicine, Seoul National University College of Medicine, Seoul 03080, Republic of Korea; 5 Department of Plastic Surgery and Institute of Aesthetic Medicine, CHA Bundang Medical Center, CHA University, Seongnam 13496, Republic of Korea; 6 Department of Surgery, Seoul National University Hospital and College of Medicine, Seoul 03080, Republic of Korea; 7 Department of Psychiatry, Kangwon National University, Chuncheon 24289, Republic of Korea; o2pamu@naver.com; 8 Unidocs Inc., Seoul 03080, Republic of Korea; tobewisey@unidocs.co.kr; 9 Department of Psychiatry, Chungnam National University Hospital, Daejeon 30530, Republic of Korea; iris870422@gmail.com (S.Y.J.); thomasign@gmail.com (J.L.K.); 10 Department of Psychiatry, Chungnam National University College of Medicine, Daejeon 30530, Republic of Korea; 11 Department of Psychiatry, Healthcare System Gangnam Center, Seoul National University Hospital, Seoul 03080, Republic of Korea; gong@snuh.org

**Keywords:** Alzheimer’s disease, mental status and dementia tests, early diagnosis, speech acoustics, deep learning, digital healthcare

## Abstract

Background: Alzheimer’s disease (AD) is the most common form of dementia, which makes the lives of patients and their families difficult for various reasons. Therefore, early detection of AD is crucial to alleviating the symptoms through medication and treatment. Objective: Given that AD strongly induces language disorders, this study aims to detect AD rapidly by analyzing the language characteristics. Materials and Methods: The mini-mental state examination for dementia screening (MMSE-DS), which is most commonly used in South Korean public health centers, is used to obtain negative answers based on the questionnaire. Among the acquired voices, significant questionnaires and answers are selected and converted into mel-frequency cepstral coefficient (MFCC)-based spectrogram images. After accumulating the significant answers, validated data augmentation was achieved using the Densenet121 model. Five deep learning models, Inception v3, VGG19, Xception, Resnet50, and Densenet121, were used to train and confirm the results. Results: Considering the amount of data, the results of the five-fold cross-validation are more significant than those of the hold-out method. Densenet121 exhibits a sensitivity of 0.9550, a specificity of 0.8333, and an accuracy of 0.9000 in a five-fold cross-validation to separate AD patients from the control group. Conclusions: The potential for remote health care can be increased by simplifying the AD screening process. Furthermore, by facilitating remote health care, the proposed method can enhance the accessibility of AD screening and increase the rate of early AD detection.

## 1. Introduction

Alzheimer’s disease (AD), the most common form of dementia, is a neurodegenerative disease characterized by cognitive decline [1,2,3]. Among the various causes of dementia, AD accounts for 60–70% of all cases [4]. The increase in life expectancy has been associated with a steady increase in the population with AD [5,6]. After the age of 65, the likelihood that a person will develop AD doubles every five years. Therefore, the number of patients with dementia will reportedly be over three times higher by 2050 compared to 2010 [7,8].

From the early stages to the most severe cases, the symptoms of AD include decreased spatial awareness, lack of concentration, and distraction [9,10]. Other major symptoms include memory impairment and deficits in language skills [11,12,13]. Additionally, physical function decreases, which makes it difficult to perform daily activities; therefore, patients lose autonomy and become dependent on others for care [13,14]. However, deterioration in language ability can impair communication, thereby making daily life difficult for AD patients and caregivers [14,15]. Over time, family members of AD patients may experience increased physical and emotional exhaustion [16,17]. Moreover, owing to the high costs of all care, diagnosis, and pharmacological treatment, AD is one of the most expensive chronic diseases [15,18]. For example, the cost of caring for patients with AD and other forms of dementia is over twice that of patients of the same age suffering from cancer and 74% higher than those with cardiovascular diseases [19,20]. Consequently, patients and their families incur a financial burden [14,16,17,18].

Currently, there is no cure for AD, and it is considered a very serious disease [2,4]. However, if detected early, the progression of symptoms can be delayed or alleviated with medication [21,22]. A definitive diagnosis of AD includes diagnostic techniques such as genetic tests, cerebrospinal fluid tests, positron emission tomography (PET), and magnetic resonance imaging (MRI), which can be costly and invasive [23,24]. Therefore, they are unsuitable for early diagnosis [18,23,24]. In addition, various standards for AD diagnosis exist, but most depend on the results of tests performed by experts [25,26]. Furthermore, AD has social consequences, such as the cost to the national economy. Therefore, there is increasing interest in the development of simple screening techniques that can provide an easy and convenient diagnosis that is accessible and low-cost [27,28,29].

One possible solution is using speech analysis and processing to detect changes in language ability, which can facilitate the early detection of AD [30,31]. These changes could be a key indicator for the preclinical stages of AD [12,30] and for patients experiencing greater difficulty speaking as the disease progresses [13]. AD patients may show speech-related symptoms such as hesitation, frequent pauses, blurred pronunciation, tremors, light stuttering, the use of irregular words, reduced verbal fluency, changes in the rhythm of speech, deviation from simple grammatical and lexical rules, slow or irregular breathing, and an inability to control breathing [12,31]. Moreover, there is a close relationship between language ability and cognitive ability [32,33]. These characteristics can be used as an initial indicator to distinguish between AD-related anomic aphasia and non-AD pathology [12,30]. In terms of the order of symptom manifestation, it can be considered that language impairment occurs before memory impairment; therefore, it can be a good predictor of early AD [32,33].

Although changes in acoustic and vocal rhythm may be imperceptible to the human ear, advances in automatic speech analysis technology have made it possible to identify and effectively extract these acoustic and temporal parameters [14,34]. Speech biometrics or automatic speech analysis are considered ideal tools for assessing cognitive deficits or changes in older adults, as these methods are capable of recording speech planning, sequencing, and performance in real time [35]. Recently, various studies have attempted to classify spoken language using different speech processing techniques and algorithms to identify the early signs of cognitive decline [14,27,35,36].

In addition to the symptoms of language disorders, the vocabulary level, complexity of syntactic structure, and use of irregular words are significantly affected by factors such as age, educational background, and cognitive ability; therefore, it is difficult to use these predictors as indicators of early AD [37]. In contrast, the frequency of hesitation, impaired affective prosody, emphasis of specific syllables, changes in tempo or timing, differences in pitch and intonation, and irregular breathing can be used as indicators in speech analysis and processing of voice signals [38,39,40]. Language analysis is important owing to its suitability for classification; some studies have shown that it can be used to distinguish between people with and without AD with over 91.2% accuracy [41,42].

The COVID-19 pandemic has increased the demand for remote diagnosis and management of AD [43,44]. The existing mode of mini-mental state examination for dementia screening (MMSE-DS) requires patients to visit medical institutions for in-person screenings. Therefore, the restrictions imposed due to the COVID-19 pandemic have made early AD diagnosis difficult [45,46]. To satisfy the changing requirements, government agencies are planning to introduce a remote AD management system that will enable elderly people with reduced mobility to undergo dementia screening examinations at home [47,48,49]. Hence, there is a pressing need to develop measures with greater accuracy and efficiency for remote AD diagnostic screening [48,50,51]. Previously, remote dementia management systems were implemented over the phone [29,32,35]. Also, in recent years, there has been active research on the delivery of telemedicine via smart devices and applications [45,46]. Various smart devices are highly suitable for the diagnosis and remote management of AD, considering they can quickly and easily capture voice and image data and, in some cases, record basic bio-signals [46,49,52].

In this study, MMSE-DS is performed with AD patients and healthy adults to select factors that are significant for classifying dementia patients based on case records. After pre-processing the voice data obtained for each item, mel-frequency cepstral coefficients (MFCCs) are used to produce a spectrogram by arranging the coefficients in a specific order defined by the authors and synthesizing them into a single image. These images are used for training and are applied to different deep learning models to obtain high accuracy. Furthermore, we establish the criteria for the selection of factors suitable for analysis based on the MMSE-DS and voice data. Lastly, we verify that the proposed method can be used to diagnose AD with high accuracy compared to MMSE-DS by utilizing voice signals that can be easily acquired by exchanging simple questions and answers online using deep learning methods without establishing a special examination system for AD screening.

## 2. Methods

### 2.1. Patient Information

The voice data of AD patients (experimental group) and healthy adults (control group) were obtained by applying MMSE-DS to 88 adults aged 50–75 years who expressly indicated their voluntary intention to participate (Table 1). The study was conducted at Chungnam National University Hospital between 1 April 2019 and 23 December 2019. The experimental group included those who had been diagnosed with AD in the last three months and those who had a clinical dementia rating between 0.5 and 2. 

All experimental groups were confirmed to be AD patients through MRI, blood tests, and neuropsychological tests. Patients with a rating > 3 or those who were unable to undergo screening were excluded from this study. The control group was recruited via a participant recruitment notice, and those who were deemed capable of conducting the screening test were identified through a simple interview with a specialist. Finally, 42 patients who scored at least 26 points were selected as the control group.

This study was approved by the institutional review board of Chungnam National University Hospital (human subject study, prospective study, observational study, controlled study) (IRB approval number: CNUH2019-02-068), and the study protocol adhered to the ethical guidelines of the 1975 Declaration of Helsinki. The MMSE-DS for all participants was performed by one trained clinical psychologist. The entire MMSE-DS process for each participant was recorded as a video and saved as an MP4 file. Out of the 88 participants recruited, eight were excluded because they expressed their wish to withdraw. Of the remaining 80 participants, 24 were men (11 in the experimental group) and 56 were women (29 in the experimental group), i.e., accounting for 30 and 70% of the total participants, respectively. The mean age of the participants was 68.8 years. The average length of education was 9.36 years; 11.2 years for men and 8.27 years for women.

### 2.2. Clinical Data Collection

The screening test was conducted using MMSE-DS, the most commonly used AD screening method by public health centers in South Korea. MMSE-DS includes the following elements: temporal orientation, spatial orientation, memory registration, attention, recall, visual denomination (naming), following a three-stage command, phrase repetition, visuospatial construction, reading, writing, comprehension, and judgment, and analysis of scores [53,54,55]. Considering this study only used voice characteristics, the results for the questions that required spoken answers were used [56,57]. Table 2 summarizes the MMSE-DS composition and questions.

Considering this study aims to perform a classification of AD patients by analyzing voice data, we assumed that it would be advantageous and efficient to exclude some of the results instead of using them all [33,35,36,57]. The selection of the questionnaires was based on the expert advice of a focus group interview conducted by psychiatrists at Chungnam National University Hospital. The questionnaires recommended excluding some questions on temporal orientation, the ability to follow a command, spatial orientation, and visuospatial construction as they required judgment, visual denomination, and abstract thinking. In particular, questions on the ability to follow a command and visuospatial construction did not require a verbal response; hence, they were not suitable for this study [32,58].

Next, based on the experts’ advice, items such as spatial orientation, temporal orientation, judgment, and visual denomination were gradually excluded to see the results. Finally, twelve questions were determined to be most suitable for distinguishing AD patients from the control group, which are marked with an asterisk in Table 2. They include three items for memory registration, five items for attention and calculation, one item for delayed recall, and one item for temporal orientation [53,54,59,60]. Although the responses to all 28 questions were obtained, only twelve results were used for the subsequent MFCC deep learning and spectrogram.

MMSE-DS was performed once per participant, and the voice responses were acquired for 88 participants. All the recorded data were valid. After screening, eight patients expressed their intention to withdraw from the study, and their results were excluded. Once the consent of the remainder of participants was obtained, the video and audio data were recorded for the remaining 80 participants using a webcam (Logitech BRIO 4K webcam) with supported 30 fps, ultra-high definition, and 4096 × 2160 resolution.

Among the recorded voice responses, the parts corresponding to the participants’ answers were edited and saved as a wave file using the Cubase audio editor.

### 2.3. Data Preprocessing

**Figure 1:**
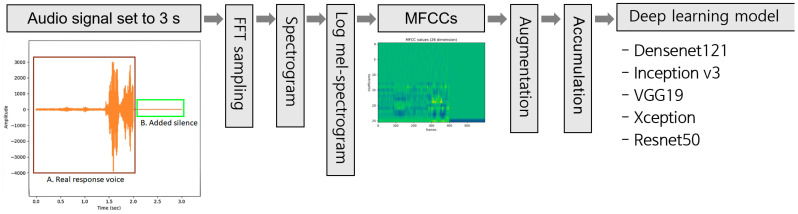
Overall process flow of the study.

The audio data corresponding to the responses to the twelve selected questions was extracted from the MMSE-DS videos using the ffmpeg tool with a sampling rate of 44.1 kHz and a stereo channel format. Then, the MP4 file format was used to minimize information loss in the audio. The audio response information was set to 3 s as the participants’ answers were completed within 1–2 s in most cases. When the response was completed in less than 3 s, silence was added to create a file with a total length of 3 s. Participants in the AD group were often unable to answer the questions. In this case, they were given 30 s; if there was still no answer, the next question was asked. If a participant could not answer the questions, the entire 30 s period was treated as silence. In these cases, when the voice data was converted to a spectrogram, the length of the dataset became long without containing significant meaning. Therefore, to construct efficient training datasets, a 3 s clip of the 30 s silence was extracted and converted to a spectrogram. As all participants responded within 1–2 s, “no response”, which was expressed as 3 s of silence, was sufficiently distinguishable. Features from one wave file were converted to one image using the MFCC, and twelve MFCC images were extracted from twelve wave files. Next, these images were combined and reconstructed to form one image file. Figure 1 shows a conceptual diagram of the pre-processing procedure.

### 2.4. Spectrogram

**Figure 2:**
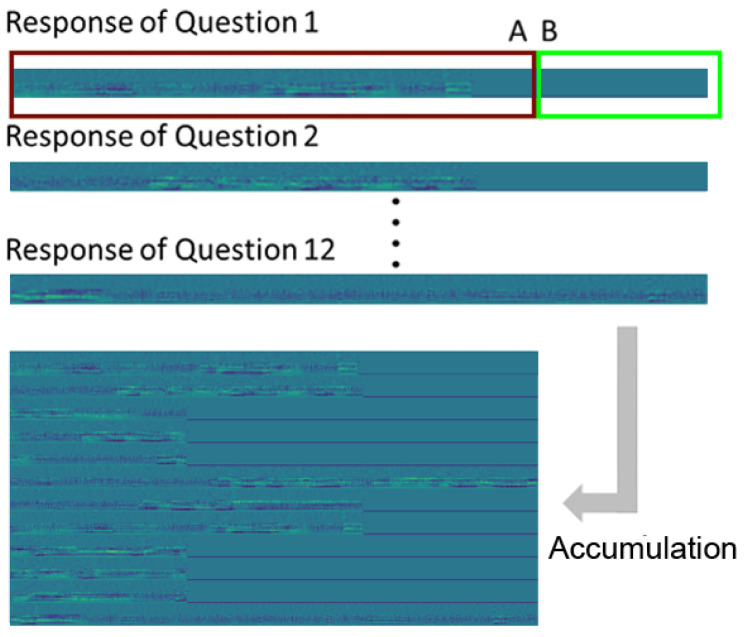
MFCC image generation of each participant’s response. (**A**) The answers to each question were standardized to a duration of 3 s. (**B**) If there was no answer at the end of this period, silence was added to the entire duration.

Extracting features from a wave file using MFCCs is a common technique used when processing voice signals [61,62]. The sampling rate of the wave file was 44.1 kHz, which indicated that there were 44,100 signals s^−1^. Each wave file had a duration of 3 s, which gives 132,300 signals per file. If 0.025 s of audio information is defined as one frame of the wave file, then there are 1103 signals per frame (rounded to the nearest whole number). The period of frame extraction was 0.01 s; hence, the frame information was extracted by skipping 0.01 × 44,100 = 441 signals. Therefore, if MFCC information was extracted from 132,300 signals for 3 s, a width of 599 MFCC images was obtained. The MFCC image was 26 pixels high because 13 MFCC feature values and 13 MFCC first derivative values were extracted from each frame. Therefore, the MFCC image generated for each 3 s response wave file was 599 × 26 pixels. By compiling the results for all twelve questions on the vertical axis, the learning data (MFCC) comprising 599 × 312 pixels were generated for each participant’s response. This description is shown in Figure 2.

### 2.5. Deep Learning Algorithms

Figure 1 shows the overall process, wherein the MFCC images are generated from the audio data and used to train and test deep learning models. To improve the accuracy during training, ten-fold data augmentation was performed by translating the data horizontally and changing the brightness [63]. Because speech characteristics are expressed as patterns and textures in MFCC images, augmentation methods that induce morphological transformation were avoided. Further, we experimentally examined the range in which the change in brightness value in MFCC image augmentation is suitable for learning in deep learning. The brightness was changed by 80–120% compared to that of the original image, in 5% increments. The “horizontal shift” led to a delayed response effect, and the changes according to the shift range were validated and applied so that the effect was not excessive. The horizontal shift was increased to ±25% in 5% increments.

In order to perform accurate augmentation, we confirmed the section where the Densenet121 model showed the highest performance after augmenting data for brightness and horizontal shift, respectively. As the convolutional network incorporates shorter connections between layers closer to the input and those closer to the output, the network becomes deeper, more accurate, and capable of learning more efficiently. Densenet121 connects each layer to every other layer in a feed-forward manner. In traditional convolutional networks, each layer has L connections (one connection to the next layer), whereas Densenet121 has L × (L + 1)/2 connections. For each layer, all previous feature maps are used as inputs, and its own feature map is utilized as inputs for all subsequent layers. Therefore, the advantages of Densenet121 include: (1) alleviating the vanishing-gradient problem and enhancing feature propagation. (2) encouraging feature reuse and reducing the number of parameters. Considering these characteristics, the Densenet121 model, which is suited for MFCC analysis, was selected in this experiment [64,65]. Through this process, we performed data augmentation techniques that were advantageous for deep learning training. The corresponding results are presented in Appendix A (Table A1 and Table A2). Based on these results, we finally applied brightness ±15% and right shift 20% augmentation to our training dataset. The eighty MFCC images obtained from the participants were augmented tenfold; hence, the results comprised 400 images each for healthy adults and AD patients.

To classify healthy adults and AD patients using MFCC images, training and predictions were performed using five deep learning models, which are representative CNN algorithms for image classification and include Densenet121, Inception v3, VGG19, Xception, and Resnet50 [66]. In the MFCC image, the signal characteristics were expressed in the form of a pattern or texture. Therefore, deep learning models suitable for image pattern or texture type classification were selected [67,68,69]. The performance of the five CNN algorithms was evaluated using the five-fold cross-validation and hold-out methods, wherein the datasets were divided in a ratio of 8:1:1 for training, validation, and testing, respectively [70].

The training for each algorithm was stopped early to avoid overfitting by checking the training and validation losses three times. AdaMax was used as an optimizer to optimize the deep learning models, with a learning rate of 1 × 10^−6^ and a batch size of 4. For all five types of models, the number of epochs was equally applied to 60, the number of training data was 640, and the length of the test data was 16. In the case of five-fold cross-validation, all models were iterated five times and the validation splits were set at 0.1. 

## 3. Results

### Performance Comparison

**Figure A1:**
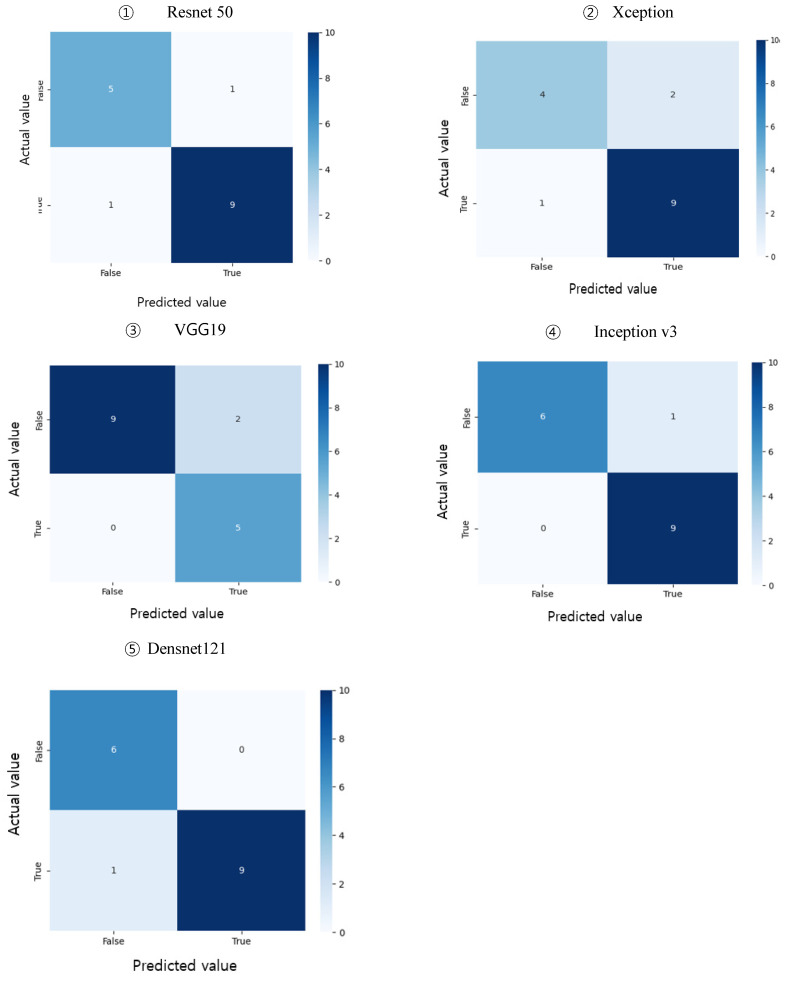
The confusion matrix for each algorithm of the Hold-out method.

**Figure A2:**
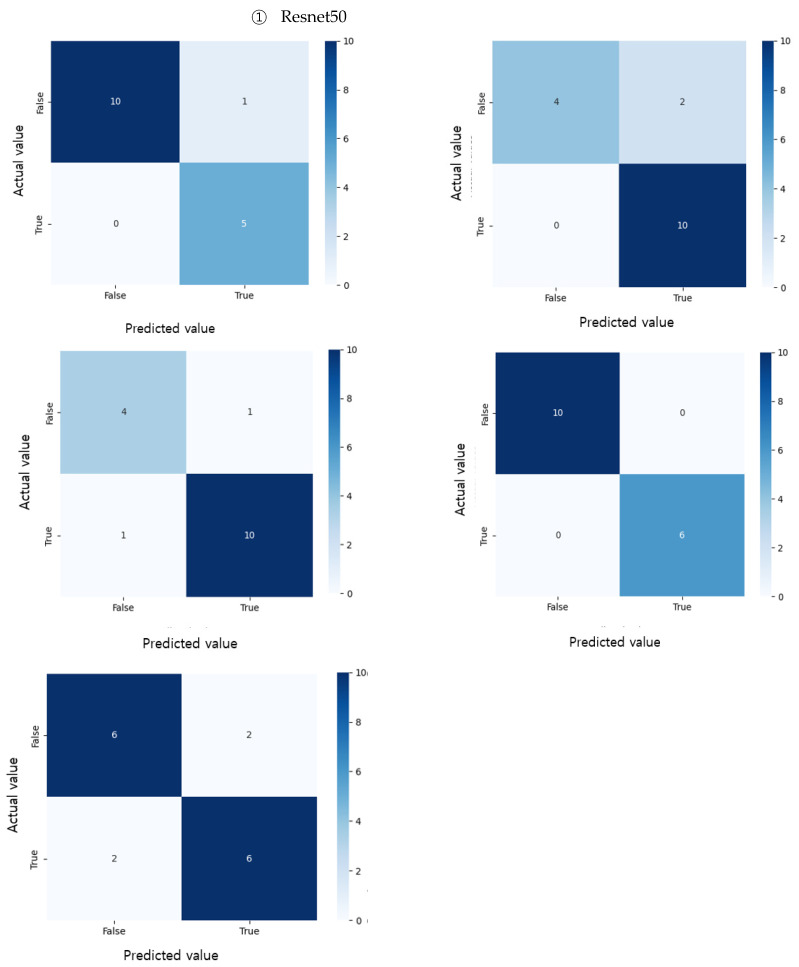

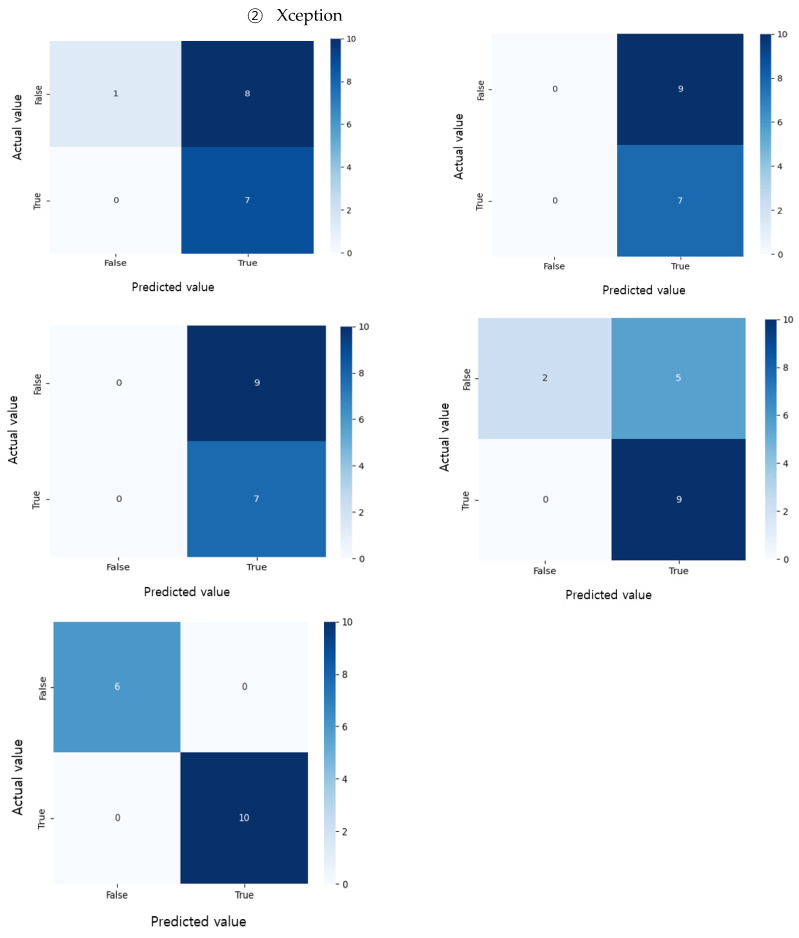

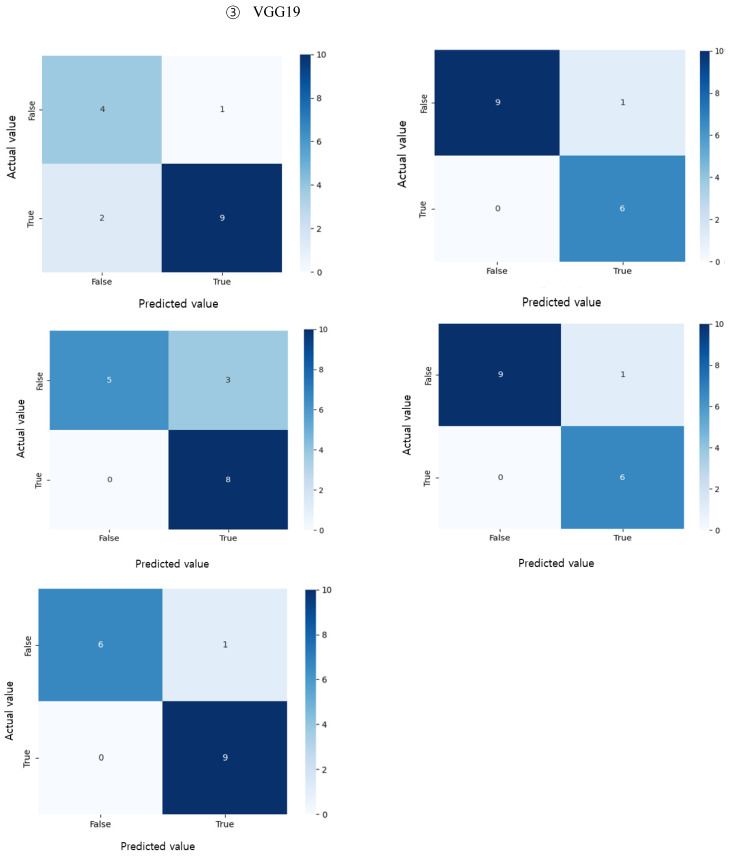

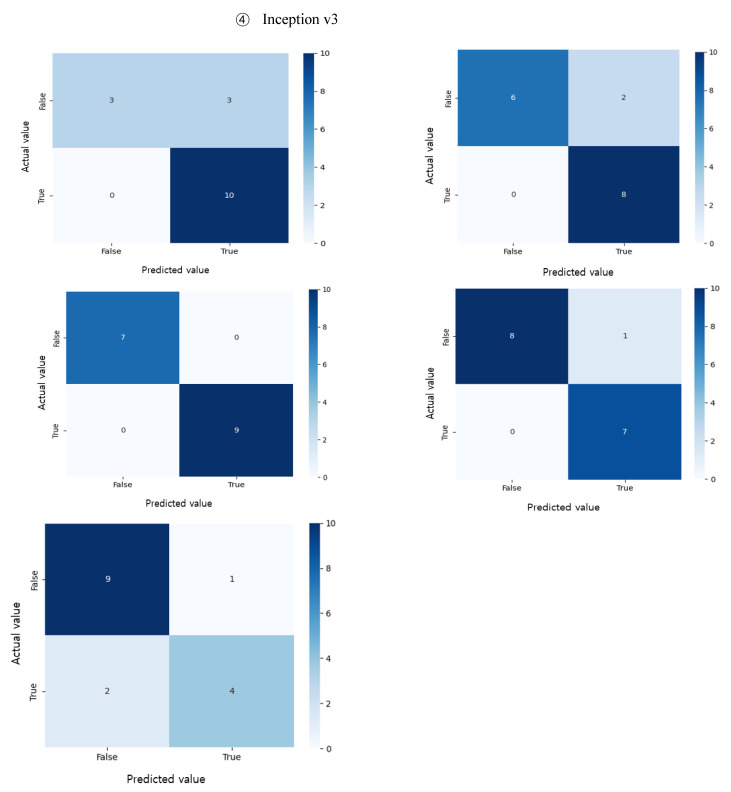

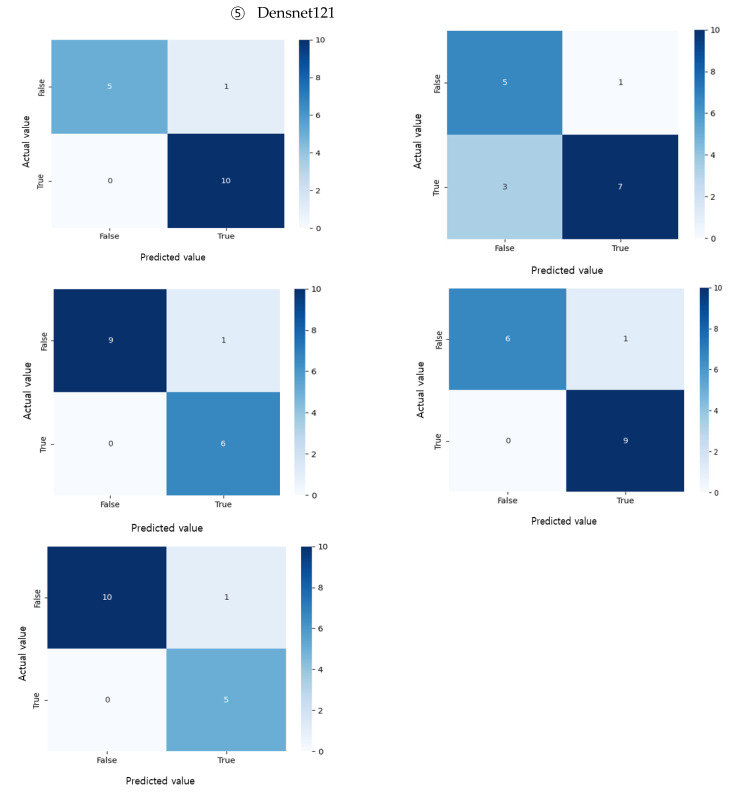
The confusion matrix for each algorithm of the Five-fold method.

In order to acquire accurate results for the five deep learning algorithms, the performance results of the hold-out and cross-validation methods were obtained. In this experiment, we focused on metrics such as sensitivity, which is the value predicted by the model as AD patients among actual AD patients, and positive predictive value (PPV), which represents the ratio of actual AD patients among predicted AD patients [71]. Additionally, the F1-score was utilized to assess the model’s balanced performance between positive and negative predictions. These metrics provide valuable insights into the performance of the classification model in terms of correctly identifying positive instances, the accuracy of positive predictions, and the overall balance between precision and recall. Furthermore, metrics such as specificity and negative predictive value (NPV), which is a value representing the actual proportion of normal people among normal people, were calculated and shown in Table 3 and Table 4. The confusion matrix for each algorithm is in Figure A1 and Figure A2 of Appendix A.

**Figure 3:**
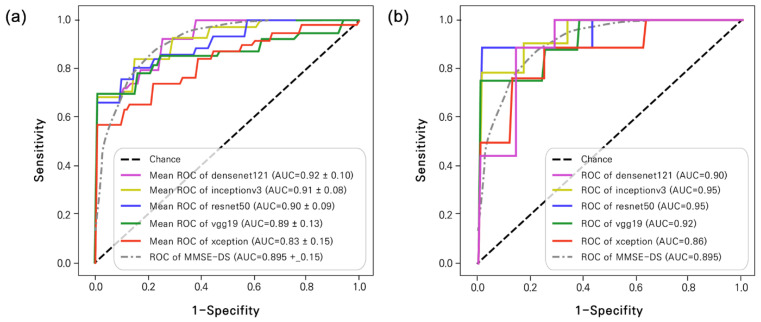
Receiver operating characteristic (ROC) results. (**a**) Five-fold cross-validation and (**b**) Hold-out methods. The gray line indicates the AUC of MMSE-DS [60].

As a result, in the five-fold cross-validation, Densenet121 showed the highest performance with a sensitivity of 0.9550, an accuracy of 0.9000, a PPV of 0.8791, an F1-score of 0.9139, and an AUC of 0.9243. As shown in the AUC graph (Figure 3), Resnet50 and Inception v3 also showed high performance. Likewise, in the results of the hold-out validation, Densenet121, Inception v3, and Resnet50 showed high performance in terms of sensitivity, accuracy, PPV, F1-score, and AUC. Figure 3 compares the results obtained using the hold-out and five-fold cross-validation methods. The gray dashed curve represents the AUC of MMSE-DS [59].

## 4. Discussion

This study presented a method of screening AD patients using only voice data. Voice data were collected, pre-processed, normalized, and converted into a spectrogram to obtain MFCC images. Deep learning models are commonly trained with the MFCC transition to utilize the advantages of sound signals and non-verbal elements [71,72,73,74]. MFCC features have been widely used in voice classification tasks, as they have been shown to perform well in terms of robustness and discrimination power. These features are based on the mel-frequency scale, which is a non-linear frequency scale that is closely related to the non-verbal elements. For this reason, we used MFCC and two-dimensional spectrogram images [75,76,77]. The MMSE-DS assessment tool, the most widely used method for interviewing AD patients in public health centers in South Korea, was used to acquire the voice data. The MMSE-DS comprised 28 questions that were used to screen patients for their attention and calculation, temporal orientation, memory registration, and delayed recall. 

Responses were obtained for all questions with MMSE-DS. At the beginning of the experiment, we trained the deep learning model using all the questions. We attempted to train the model in various ways, but the ROC value was very low at 0.6. Therefore, we judged that it is more effective to select valid questions and use them for model training than to use all questionnaires. For this reason, not all of these responses were converted into MFCC images; only 12 questions were finally selected based on expert advice from a focus group interview conducted by psychiatrists at Chungnam National University Hospital. The excluded questions were based on temporal orientation, ability to follow a command, spatial orientation, and visuospatial construction, as they required judgment, visual denomination, and abstract thinking. In particular, the ability to follow a command and visuospatial construction questions did not require a verbal response, and hence, they were not suitable for this study [32,58].

The answers to each question were standardized to a duration of 3 s. One MFCC image was generated, and the answers to all 12 questions were compiled to generate a training image. The responses were standardized to 3 s because none of the responses in either group exceeded this duration. However, the AD patients were unable to answer some questions, in which case a waiting time of 30 s was provided. If there was no answer at the end of this period, silence was added to the entire duration. The texture of the MFCC image of the part processed as silence was different from the voice signals, and if all 30 s of the wait time were used, the ratio of the texture of silence would become unnecessarily high. Considering that these factors hinder learning performance, the voice data were standardized to 3 s, which was found to be an appropriate time to receive an answer.

In this study, the AD classification accuracy of the deep learning model was measured using the five-fold cross-validation and hold-out validation methods. In the five-fold cross-validation method, the Densenet121 model exhibited the highest overall accuracy. Inception v3 and VGG19 also exhibited high accuracy. In the hold-out validation method, Resnet50, Inception v3, and Densenet121 showed high performance. 

The conventional methods used for a definitive diagnosis of AD, such as genetic tests, cerebrospinal fluid tests, PET, and MR imaging, are invasive and costly, making them less accessible [23,24]. Moreover, these methods typically require expert interpretation [25,26]. In contrast, the approach presented in this study, utilizing a deep learning model based on voice analysis, offers a non-invasive, cost-effective, and time-efficient alternative. If it is more systematized, it may be automated and utilized without the help of experts.

Overall, the Densenet121, Inception v3, resnet50, and VGG19 models performed excellently and exhibited similar or superior performance to the MMSE-DS [59]. When the voice signals of the responses were analyzed to classify the AD patients, we achieved a sensitivity of 0.9550, a specificity of 0.8333, an accuracy of 0.9000, and an area under the curve (AUC) of 0.9243 in the five-fold cross-validation method. The accuracy of this study, wherein the spectrogram of the voice data was used to train a convolutional neural network (CNN) to classify AD patients, was higher than that reported by Duc and Ryu (85.27%), who investigated the correlation between the 3D-functional MRI results and MMSE scores [78], and Tae Hui Kim (0.895), who evaluated the diagnostic accuracy of MMSE-DS [59]. Additionally, this study showed greater accuracy than the results reported by Liu and Cheng (91.2%), who used FDG-PET images to classify AD patients using a CNN and a recurrent neural network [79].

Considering AD patients were classified solely based on the analysis of voice signals, questions from the MMSE-DS requiring execution, visuospatial construction, judgment, and abstract thinking were not included in the experimental data based on recommendations from psychiatrists. This study aimed to show that AD classification is possible without these elements, and hence, the results are significant. In addition, it is considered meaningful to confirm which questionnaire among the MMSE-DS is suitable for voice-based deep learning classification. The academia community related to AD in South Korea judged that these results were reliable and useful for screening AD patients. In this study, an individual effect analysis of each question was not performed. It is expected that more diverse and accurate results can be acquired if the scope is expanded by performing an individual analysis of each question, a validation of a combination of various questions, and an analysis including additional elements. 

We have tried to detect Alzheimer’s disease based on the audio data without including any semantics. Therefore, we focused on non-verbal characteristics in the responses of patients with Alzheimer’s disease, and for this purpose, we recruited subjects and designed an experiment. Actually, the experiment’s results showed that Alzheimer’s dementia patients had distinctive features in intonation and nuance, as well as inaccurate pronunciation, a slow pace, and the elongation of vowel sounds, regardless of whether the answer was correct or not [71,72,73,74].

As this was an experimental study in which real AD patients were recruited, there are limitations in terms of the subjects and data. Because the experiment was conducted with a limited number of 80 people, this study could not perform external validation. In future studies, subject recruitment will be conducted on a large scale, and subjects from external institutions for external validation will be considered. In addition, other voice data, not responses to specific answers, will be collected, compared, and analyzed. Also, the severity of dementia will be classified using a deep learning model.

With respect to data augmentation, there are concerns that traditional data augmentation methods (flip, crop, enlargement, reduction, rotation, inversion, etc.) may negatively impact patterns or textures reflecting non-verbal characteristics in MFCC images. As can be seen in Table A1 and Table A2, the augmentation range of the brightness and horizontal shift also preserves the quality of usable data by validating the effective range. It is expected that more accurate and valid results can be obtained by increasing the number of participating subjects and the amount of data.

From a clinical point of view, our results have several strengths. First, MMSE-DS requires checking all 28 items, while our results require only 12 items, making it simple and time-saving. Second, the MMSE-DS should have different cut-off scores according to educational background, age, and gender. It is convenient and useful because clinicians do not have to consider various conditions for dementia screening.

But there are also limitations. Dementia occurs when there is a severe decrease in function in some of the various cognitive domains. Since this study used only temporal orientation, memory registration, delayed recall, and attention, the evaluation of other cognitive functions may be limited. However, as mentioned earlier, in the early stages of dementia, generally there is a decrease in peripheral awareness, a lack of concentration, distraction, memory impairment, and a decrease in language ability. In other words, if early dementia patients can be selected through voice and responses to limited items, it is expected to bring about a fundamental innovation in the dementia screening method.

This study is a simplified dementia screening test, so further validation of each question’s reliability is needed, but it is considered to be a useful technique for screening high-risk groups. It will also be of great help in developing a platform that performs high-risk screening, precise diagnostic testing, and management.

If the deep learning model proposed in this study is used, AD screening can be performed more easily and quickly. Based on this, it is possible to build a telemedicine or screening automation system through smart devices [46,52]. By installing them in an easily accessible place, the barrier to entry can be lowered, and the quality of telemedicine can be improved by utilizing virtual reality [47,48,49]. In addition, since early diagnosis of AD can inhibit its progression, it is expected that the simple screening method introduced in this paper will also work as a digital therapeutic.

## 5. Conclusions

In this study, voice data were acquired using MMSE-DS to distinguish between AD patients and healthy adults with high accuracy. In the five-fold cross and hold-out validation, Densenet121, Inception v3, and Resnet50 deep learning models showed high performance with sensitivity, accuracy, PPV, F1-score, and AUC metrics. Their performance was higher than the classification accuracy of MMSE-DS, and they also recorded good results compared to other studies that did not use voice-deep learning. Utilizing the results of this study, the screening process for AD patients can be simplified, which can contribute to increasing the accessibility of AD testing and the early diagnosis rate. In addition, it can be developed into an automated system to reduce dependence on experts and can contribute to AD screening by being applied to remote or online medical treatment.

## Figures and Tables

**Table 1:** Status of age and gender distribution related to research data.

Age	AD Patients		Healthy Adults	
	Male	Female	Male	Female
50–59	0	0	1	8
60–69	6	12	7	12
70–75	2	20	4	8
Total	8	32	12	28
	40		40	

**Table 2:** Questionnaires for the MMSE-DS used in this study.

Number	Items	Individual Questions
1-1	Temporal orientation	What year are we in now?
1-2		What is the season?
1-3		What is the date today?
1-4		What day of the week is it?
1-5 *		What month are we in now?
2-1	Spatial orientation	What city are we in?
2-2		What borough are we in?
2-3		What ‘dong’ (one of the administrative divisions) are we in?
2-4		What floor of the building are we on?
6		What is the name of this place?
10	Following a three-stage command	Please follow what I say and as it will be told only once, please listen carefully and follow accordingly.
		I will give you a piece of paper. Please take this piece of paper in your right hand, fold it in half with both hands, and place it on your lap.
11 *	Memory registration	I am going to name three objects. After I have said them, I want you to repeat them. Please remember what they are because I will ask you to name them again in a few minutes: tree (11-1 *), car (11-2 *), hat (11-3 *). Could you name the three items you have just heard?
12-1 *	Attention and calculation	What is one hundred minus seven?
12-2 *		Yes. Then, what is the result after subtracting seven from the value?
12-3 *		Yes. Then, what is the result after subtracting seven from the value?
12-4 *		Yes. Then, what is the result after subtracting seven from the value?
12-5 *		Yes. Then, what is the result after subtracting seven from the value?
13 *	Delayed recall	What are the three objects I asked you to remember a few moments ago? Tree (13-1 *), car (13-2 *), hat (13-3 *).
14-1	Visual denomination	(Showing a watch) What is this called?
14-2		(Showing a pencil) What is this called?
15	Phrase repetition	Please listen carefully to what I say and repeat accordingly. Please note that only one attempt will be allowed. Please listen carefully and repeat after I finish. Ganjang Gonjang Gongjangjang (Translation: head of the soy source factory, used for checking pronunciation)
16	Visuospatial construction (Copying interlocking pentagons)	Please see the interlocking pentagons here and copy the drawing in the following blank section.
18	Judgment	Why do you need to wash your clothes?
19		Could you explain what “many a mickle makes a muckle” means?

* Questions selected for MFCC deep learning and spectrogram.

**Table 3:** Performance metrics of the five deep learning models for the five-fold cross-validation.

Model Name	Sensitivity	Specificity	Accuracy	PPV	NPV	F1-Score	AUC
Densenet121	0.9550	0.8333	0.9000	0.8791	0.9314	0.9139	0.9243
Inception v3	0.9305	0.8099	0.8750	0.8556	0.9179	0.8887	0.9177
VGG19	0.9750	0.8236	0.9000	0.8494	0.9778	0.9013	0.8886
Xception	0.9455	0.3183	0.6000	0.5855	0.9143	0.6997	0.8349
Resnet50	0.8944	0.8979	0.9000	0.8994	0.9042	0.8956	0.9286

**Table 4:** Performance metrics of the five deep learning models for the hold-out validation.

Model Name	Sensitivity	Specificity	Accuracy	PPV	NPV	F1-Score	AUC
Densenet121	1.0000	0.7143	0.8750	0.8182	1.0000	0.9000	0.9048
Inception v3	0.9000	0.8333	0.8750	0.9000	0.8333	0.9000	0.9500
VGG19	1.0000	0.6250	0.8125	0.7273	1.0000	0.8421	0.9219
Xception	1.0000	0.2500	0.6250	0.5714	1.0000	0.7273	0.8594
Resnet50	0.8889	1.0000	0.9375	1.0000	0.8750	0.9412	0.9524

## Data Availability

Data was evaluated in the process of the manuscript reviewing (https://github.com/mikeahn00/ahn_experiment), but I concerned the data open access due to privacy.

## Abbreviations

AD, Alzheimer’s disease; AUC, area under curve; CNN, convolutional neural network; MFCC, Mel-frequency cepstral coefficient; MMSE-DS, mini-mental state examination for dementia screening; MRI, magnetic resonance imaging; NPV, negative predictive values; PET, positron emission tomography; PPV, positive predictive values; ROC, receiver operating characteristic; t-SNE, t-distributed stochastic neighbor embedding.

## Appendix A

**Table A1:** The augmentation range of the brightness.

Model Name	Augmentation	Sensitivity	Specificity	Accuracy	PPV	NPV	F1-Score	AUC
Densnet121	Brightness ±5%	1.0000	0.8571	0.9375	0.9000	1.0000	0.9473	1.0000
Densnet121	Brightness ±10%	1.0000	0.8000	0.8750	0.7500	1.0000	0.8571	0.9666
Densnet121	Brightness ±15%	1.0000	1.0000	1.0000	1.0000	1.0000	1.0000	0.9682
Densnet121	Brightness ±20%	1.0000	0.8888	0.9375	0.8750	1.0000	0.9333	0.9523

**Table A2:** The augmentation range of the horizontal shift.

Model Name	Augmentation	Sensitivity	Specificity	Accuracy	PPV	NPV	F1-Score	AUC
Densnet121	Right shift 5%	1.0000	0.8750	0.9375	0.8888	1.0000	0.9411	0.9375
Densnet121	Right shift 10%	1.0000	0.8571	0.9375	0.9000	1.0000	0.9473	0.9365
Densnet121	Right shift 15%	1.0000	0.9000	0.9375	0.8571	1.0000	0.9230	0.9833
Densnet121	Right shift 20%	1.0000	1.0000	1.0000	1.0000	1.0000	1.0000	1.0000
Densnet121	Right shift 25%	0.8571	1.0000	0.9375	1.0000	0.9000	0.9230	1.0000

The confusion matrix for each algorithm is in Figure A1 and Figure A2.
